# REDUCING HOSPITAL ADMISSIONS FOR PEOPLE LIVING WITH DEMENTIA—RESULTS FROM THE GERMAN DEMWG-STUDY

**DOI:** 10.1093/geroni/igad104.0767

**Published:** 2023-12-21

**Authors:** Karin Wolf-Ostermann, Annika Schmidt, Susanne Stiefler, Janissa Altona, Andre Kratzer, Carolin Donath, Elmar Graessel

**Affiliations:** University of Bremen, Bremen, Bremen, Germany; University of Bremen, Bremen, Bremen, Germany; University of Bremen, Bremen, Bremen, Germany; University of Bremen, Bremen, Bremen, Germany; Universitätsklinikum Erlangen, Erlangen, Bayern, Germany; Universitätsklinikum Erlangen, Erlangen, Bayern, Germany; Psychiatrische Universitaetsklinik Erlangen, Erlangen, Bayern, Germany

## Abstract

People living with dementia (PlwD) have a high risk of being admitted to hospital, especially in advanced stages of dementia – with often severe negative consequences. The DemWG-study therefore aimed to reduce the number of hospital admissions for long-term care dependent PlwD by a complex intervention - including motor and cognitive training for PlwD and education of caregivers and general practitioners. By means of a cluster randomised longitudinal design, data on hospital admissions as well as quality of life, risk of falls, challenging behaviours and cognitive functioning were assessed in 97 shared-housing arrangement (SHA) in Germany with a total of 341 PlwD at baseline and after a 6 months intervention. Results of the of longitudinal data analysis showed a significant decline in hospital admissions for the intervention period and less challenging behaviours. Quantitative and additionally qualitative data were also collected 12 and 18 months after baseline. Caregivers perceived improved social interaction, which in turn has a positive effect on living together in the SHA. Based on the results and in light of the urgent need for easy to implement non-pharmacological and/or psychosocial therapy approaches recommendations for better care and support can be derived and the intervention can be implemented in everyday practice.

